# Dispositional factors in the explanation of symptoms of depression, anxiety, health anxiety and COVID-19 Phobia

**DOI:** 10.1371/journal.pone.0299593

**Published:** 2024-04-16

**Authors:** Eric Mayor, Roselind Lieb

**Affiliations:** Division of Clinical Psychology and Epidemiology, Department of Psychology, University of Basel, Basel, Switzerland; Julius-Maximilians-Universität Würzburg: Julius-Maximilians-Universitat Wurzburg, GERMANY

## Abstract

Maladaptive personality, the motivational systems, and intolerance of uncertainty play important roles in the statistical explanation of depression and anxiety. Here, we notably examined for the first time whether symptoms of depression, anxiety, health anxiety, and fear of COVID-19 share similar associations (e.g., variance explained) with these important dispositional dimensions. For this cross-sectional study, data from 1001 participants recruited in Germany (50% women; mean age = 47.26) were collected. In separate models, we examined the cross-sectional associations of the symptoms of depression, anxiety, health anxiety, and fear of COVID-19 with the Personality Inventory for DSM Short Form Plus scales, the Behavioral Inhibition System / Flight–Fight–Freeze System / Behavioral Activation System scales, and Intolerance of Uncertainty scales. Relative weight analyses were used to determine the within-model importance of the different scales in the prediction of the symptoms. All in all, our study showed that maladaptive personality and intolerance of uncertainty dimensions are more important sets of predictors of the studied outcomes (with which depressive and anxious symptomatology feature very similar associations) than are the motivational system dimensions. Within predictor sets, the scales with the most important predictors were: Negative Affectivity, the Behavioral Inhibition System, and Burden due to Intolerance of Uncertainty. Our findings highlight the relevance of focusing behavioral targets of psychotherapy on these within-set traits and identify potential research priorities (maladaptive personality and intolerance of uncertainty) in relation to the symptoms of interest.

## Introduction

Some studies have proposed maladaptive personality (e.g., [1]), motivational systems [2], and intolerance of uncertainty (IU) [3] as important sets of dispositional constructs in the statistical explanation of symptoms of depression and, especially, anxiety. More recently, some of these associations have been investigated in the context of the COVID-19 pandemic (e.g., [4]).

More generally, in past research, these dispositional factors have been associated with internalizing (and sometimes also externalizing) symptoms (e.g., maladaptive personality: [5]; motivational systems: [6]; IU: [7]). It is considered that they constitute vulnerability to depression and, in particular, anxiety (e.g., [5–7]). Although such research has been thorough in examining the associations of different outcomes with these dispositional constructs, the different fields have evolved in isolation from each other. Thus, a gap in the literature is that the relative contribution of these dispositional factors to depression and anxious symptomatology has never been examined in one single study.

It is further worth investigating whether the relative contribution of these dispositional factors is similar or different for more specific outcomes such as health anxiety and cognition and emotion during major stressors such as COVID-19. Other studies have started examining the associations of these dispositional factors with health anxiety and fear of COVID-19 (also internalizing symptomatology), again studying each dispositional factor in isolation from the others (e.g., [4, 8–10]).

Determining which transient or durable vulnerability and protective factors have the strongest (or weakest) associations with outcomes of interest, and whether such outcomes differ in their associations with examined predictors, is an essential and frequent question in mental health research and epidemiology (e.g., [11–18]).

The main contribution of this study is to compare the explained variance of each set of predictors (e.g., maladaptive personality compared with IU in the prediction of symptoms of anxiety and depression) and to examine regularities in the role of the components within the different sets of predictors in the prediction of symptoms of the symptomatology of depression and anxiety as general outcomes. As we have mentioned, such comparisons have been overlooked in past studies, and our study addresses this research gap. Doing so, we also conceptually replicated past (rather compartmentalized) research showing that these constructs are related to symptoms of depression and anxiety (general outcomes). We also examined symptoms of health anxiety and fear of COVID-19 as more specific outcomes, which is important in a context such as the COVID-19 pandemic and informative in relation to similar potential future public health situations.

### Maladaptive personality

According to Section III of the DSM-5 ([19]), maladaptive personality domains are: negative affectivity (“Frequent and intense experiences of high levels of a wide range of negative emotions”), detachment (“Avoidance of socioemotional experience”), disinhibition (“Orientation toward immediate gratification”), antagonism (“Behaviors that put the individual at odds with other people”), and psychoticism (“wide range of culturally incongruent odd, eccentric, or unusual behaviors and cognitions”). In addition to these dimensions, the ICD-11 also includes anankastia (rigid perfectionism) ([20]). The role of negative affectivity and detachment in depressive and anxious symptomatology has been highlighted in the past [1, 21]. One particular study also examined anxiety symptoms related to the COVID-19 pandemic from this perspective [10] and found that each of the dimensions of the PID-5 (Personality Inventory for DSM-5) was related to anxiety towards COVID-19, thus demonstrating that those dimensions constitute vulnerability factors towards such specific outcomes as well. All in all, past research has established the relevance of maladaptive personality as an important predictor group of anxiety and depression, as well as of more specific health anxiety (fear of COVID-19).

### Motivational systems

Gray’s biopsychological theory is among the most influential personality theories in psychopathology research [22]. It surmises that neuropsychological differences produce durable interindividual differences in motivational systems [23–25]. There are three motivational systems in Gray’s biopsychological theory of personality (also known as reinforcement sensitivity theory [26, 27]). There are two main motivational systems according to the initial formulation of reinforcement sensitivity theory: The Behavioral Inhibition System (BIS) is related to avoidance motives and the Behavioral Activation System (BAS) to approach motives [26]. An extension of the theory also proposes the Flight–Fight–Freeze System (FFFS) (governing response to immediate threat/harm) as another main system [27]. The role of the BAS is to respond to appetitive stimuli (conditioned or unconditioned) by orienting to an approach response. The FFFS organizes conduct under aversive stimuli by orienting to an avoidance response. Finally, the BIS organizes conduct in case of conflict between avoidance and approach [27].

The motivational systems are considered relatively stable throughout life and are relevant for mental health [2]. The role of the motivational systems–the BIS, particularly–in relation to symptoms of depression and anxiety has been frequently corroborated (e.g., [2, 28, 29]). Other studies found that BAS Reward Responsiveness (BAS-RR) was associated with positive functioning and less with internalizing and externalizing symptoms, while BAS-Drive (BAS-D) was associated with externalizing symptoms [30, 31]. Cross-sectional studies showed that the BIS was related to fear of loved ones being infected with the coronavirus [32] and that the FFFS was related to intention to self-isolate in the context of the early COVID-19 pandemic [8]. In sum, past research has demonstrated that the motivational systems–the BIS and FFFS particularly–are associated with depression and anxiety as well as with fear of COVID-19 and with protective behavior during the pandemic.

### Intolerance of uncertainty

IU is a dispositional tendency to react negatively to uncertain circumstances [33]. The neural correlates of IU have been discussed in several papers, with common findings being heightened activation of the insula and prefrontal cortex in ambiguous and threatening situations, which suggests higher reactivity to negative affective situations in individuals with high IU (e.g., [34–37]). IU is, indeed, related to depressive and anxious symptomatology [3, 38–40]. Meta-analytic findings [40] show that IU is similarly associated with depression and generalized anxiety as well as obsessive-compulsive disorder, while [39] found IU to be associated with both depression and worry, albeit more strongly with worry. Worry seems more susceptible to circumstances than state anxiety, as being faced with an imminent threat increases the effect of IU on worry but not on state anxiety [38]. [3] identified differences in the associations of IU factors (general IU and inhibitory IU) with depression factors (cognitive and affective/somatic). It is worth noting that IU is related to internalizing psychopathology, but possibly not to externalizing psychopathology [41]. IU has been found to be associated with health anxiety and anxious symptomatology in relation to the COVID-19 pandemic (e.g., [4, 9, 42, 43]). [43] performed cross-lagged analyses to examine the temporal associations of IU, anxiety sensitivity, and health anxiety during the COVID-19 pandemic. They found that IU at Time 1 was significantly associated with health anxiety at Time 2, but that other cross-lagged associations were non-significant. Results in [9] showed that both IU and anxiety sensitivity incrementally predicted health anxiety. [44] showed that IU was associated with stress appraisals relating to COVID-19 and psychological distress, as well as with quality of life (negatively). In [42], IU was associated with anxiety and perceived threat in relation to the pandemic and to wellbeing (negatively), and in [36], the link between IU and wellbeing during the pandemic was found to be serially mediated by both fear of COVID-19 and ruminative thoughts. Research overall confirms the importance of IU for depressive and anxious symptomatology and has shown its relevance in relation to the COVID-19 pandemic and health anxiety more generally.

## The present study

Anxiety and major depression are frequent mood disorders; among all causes, they are the first and second contributors to disability-adjusted life-years [45]. Subthreshold symptoms of depression and anxiety are also important contributors to disability and are thus no less relevant (e.g., [46]). The understanding of the degree of association of these outcomes with the dispositional factors mentioned in the above section is our main interest here. As we collected data during the COVID-19 pandemic, we also included, as an additional and more specific outcome, symptoms related to fear of COVID-19–at the same time probably the most significant global stressor in modern history and possibly the infectious disease that claimed the most lives during the pandemic. For these reasons, therefore, health anxiety symptoms likely manifested at higher levels than usual and were included as well due to their particular relevance during the pandemic. Health anxiety symptoms lie at a level of specificity between anxiety and fear of COVID-19.

As already noted, past research has identified important dimensions that are related to such symptoms: maladaptive personality domains, the motivational systems, and IU (these will be referred to as *groups of predictors* below). However, research has not so far investigated within one single study their similarities and differences in the examination of the symptoms of the outcomes of interest. To close this research gap, this present study has sought to replicate findings from past research and has addressed three additional research questions overlooked in past research in relation to the predictors and outcomes mentioned above:

- What are the contributions of the different groups of predictors in terms of the explained variance in the examination of the different outcomes?
- What are the significant predictors in each group above and beyond the effect of the others?
- Are there similarities and differences between outcomes in terms of their most important predictor groups and predictors within groups?

## Methods

### Sample and procedure

For this cross-sectional study, we obtained a gender-balanced sample from a non-clinical population from Germany (500 men and 501 women). The mean age of participants was 47.26 years (SD = 13.94). All participants successfully passed an attention test. Socio-demographic information for this sample is presented in Table 1.

**Table 1:** Socio-demographic data.

	Group	Frequency	Percent
Gender			
	Men	500	50.0
	Women	501	50.0
Occupational status			
	Members of the legislative body, senior administrative staff and managers	32	3.2
	Academic professions/scientists	92	9.2
	Technicians and equivalent non-technical occupations	53	5.3
	Office workers and related occupations	203	20.3
	Service occupations and salesperson	123	12.3
	Skilled workers in agriculture, forestry and fishing	3	0.3
	Craft and related occupations	52	5.2
	Plant and machine operators and assemblers	14	1.4
	Auxiliary workers	11	1.1
	Members of the regular armed forces, soldiers	2	0.2
	Unemployed	50	5.0
	Student	59	5.9
	Retired	188	18.8
	Other	119	11.9
Education			
	No secondary school diploma after grade 10 or equivalent	40	4.0
	Secondary school diploma after grade 10, high school degree or equivalent	218	21.8
	Vocational (technical) school diploma, healthcare school diploma or equivalent	426	42.6
	University (of applied sciences) degree or equivalent	317	31.7

Participants were paid 3.15 euros (equivalent to approx. 3.50 USD) based on an estimated duration of 30 minutes. The recruitment of participants was conducted in early February 2022 through an online campaign by the ISO-certified panel provider Respondi among their 18 to 69-year-old panelists. We aimed to recruit a sufficient number of participants to allow the detection of small effect sizes. From the Respondi platform, participants were directed to an online questionnaire hosted on the Unipark platform. Redirects were also employed for participants to return to Respondi. The redirects were tested beforehand. The first page of the questionnaire contained an informed consent form, which the participants had to accept in order to start the survey. As such, this constituted participants’ written consent. Participants remained anonymous to the researchers. The survey was composed of the instruments described below. Participants were requested to answer all items. Drop outs were excluded. The study was approved by the local ethics review board (Ethics Committee, Department of Psychology, University of Basel).

### Measures

We used the following instruments to measure the constructs of interest. All measures have been constructed for the dimensional assessment of their constructs and had acceptable to excellent reliability in this study (see below). All scales were computed as the average value of their respective items.

#### Maladaptive personality

Maladaptive personality was measured using the German validation of the modified 36-item version of the Personality Inventory for DSM-5 Brief Form + (PID-5BF+M [47]). The PID-5BF+M reliably measures the maladaptive personality traits of the alternative model in DSM-5 and of the ICD-11. The instrument is composed in total of 18 facets (2 items per facet, rated on a four-point Likert scale) and assesses 6 domains: negative affectivity (e.g., “I worry about almost everything”), detachment (e.g., “I’m not interested in making friends.”), antagonism (e.g., “It’s no big deal if I hurt other peoples’ feelings“), disinhibition (e.g., “People would describe me as reckless”), anankastia (e.g., “Even though it drives other people crazy, I insist on absolute perfection in everything I do”), and psychoticism (e.g., “I have seen things that weren’t really there“). The reliability of the scales was good (Cronbach alpha): neuroticism, alpha = .82; detachment, alpha = .75; antagonism, alpha = .79; disinhibition, alpha = .77; anankastia, alpha = .81; psychoticism, alpha = .79.

#### Motivational systems

The motivational systems were measured using the German validation [48] of the BIS/BAS questionnaire [26]. This 20-item instrument (ratings on a 4-point Likert scale) initially assessed the BIS with 7 items. Following theoretical refinement and further factor analyses, items initially included in the Behavioral Inhibition System (BIS) scale were grouped under two subscales: the BIS (4 items; e.g., “I feel worried when I think I have done poorly on something”) and the Fight-Flight-Freeze System (FFFS; 3 items; e.g., “Even if something bad is about to happen, I rarely experience fear or nervousness”, reverse-coded) [49]. The other subscales are: the Behavioral Activation System-Reward Responsiveness (BAS-RR) subscale with 5 items (e.g., “When I’m doing well at something, I love to keep at it”), the BAS-Drive (BAS-D) subscale with 4 items (e.g., “I go out of my way to get things I want”), and the BAS-Fun Seeking (BAS-FS) subscale with 4 items (e.g., “I’m always willing to try something new if I think it will be fun.”). The reliability of the scales was acceptable to good: BIS, alpha = .81; FFFS, alpha = .72, BAS-D, alpha = .71; BAS-RR, alpha = .67, BAS-FS, alpha = .66.

#### Intolerance of uncertainty (IU)

IU was measured with the German validation [50] of the Intolerance of Uncertainty Scale (IUS, [51]), consisting of 18 items (IU-18) instead of the 27 items of the original scale (IU-27). The items are rated on a 5-point Likert scale (1 = Not representative to 5 = Completely representative). Following factor analyses, the authors of the German translation suggest 3 sub-scales: A) Limited ability to act due to IU (e.g., “When it’s time to act, uncertainty paralyses me.”), B) Burden due to IU (e.g., “My mind can’t be relaxed if I don’t know what will happen tomorrow.”), and C) Vigilance related to IU (e.g., “One should always look ahead so as to avoid surprises.”). Each subscale is composed of 6 items. The reliability of the scales was good: Limited ability to act due to IU, alpha = .89; Burden due to IU, alpha = .91; Vigilance related to IU, alpha = .81.

#### Symptoms of depression and anxiety

Symptoms of depression and anxiety were measured using the German validation [52] of the DASS-21 [53]. The instrument is composed of 3 subscales: depression (e.g., “I couldn’t seem to experience any positive feeling at all”), anxiety (e.g., “I was worried about situations in which I might panic and make a fool of myself”), and stress (e.g., “I found it difficult to relax”) and contains 21 items (7 per subscale) rated on a 4-point Likert scale. It measures the constructs of interest as experienced during the past week. Participants filled in the full instrument. Again, the reliability of the scales was good: Depression, alpha = .93; Anxiety, alpha = .89; Stress, alpha = .91.

#### Health anxiety

General health anxiety was measured using the German modification and translation [54] of the Short Health Anxiety Inventory [55]. The translation implements a modification of the initial scoring, that is, it is scored on a 5-point Likert scale ranging from Strongly disagree to Strongly agree; it contains 14 items (e.g., “I spend much of my time worrying about my health”). The period of scrutiny is either 6 months or 2 weeks. We used the 2-week version. The Cronbach alpha value for this scale was .96.

#### Fear related to COVID-19

The COVID-19 Phobia Scale [56] is a 20-item instrument which includes 4 subscales, assessing (a) psychological factors (e.g., “The fear of coming down with coronavirus makes me very anxious”), (b) psycho-somatic factors (e.g., “I experience serious stomachaches out of the fear of coronavirus”), (c) economic factors (e.g., “The possibility of food supply shortages due to the coronavirus pandemic causes me anxiety”), and d) social factors (e.g., “The fear of coming down with coronavirus seriously impedes my social relationships”). Items are rated on a 5-point Likert scale (from 1 = Strongly disagree to 5 = Strongly agree). For the purpose of this study, a bilingual translator prepared a German version of the instrument. Items were back-translated by another bilingual translator and corrected if necessary. The reliability of the scales was good: Psychological factors, alpha = .9; Somatic factors, alpha = .93; Economic factors, alpha = .89; Social factors, alpha = .89.

#### Other measures

In addition to these instruments, we asked participants to provide basic socio-demographic information as to their gender, year of birth (age was computed as 2021 minus this value), level of education, and professional status. We also included an attention check in the form of a list of possible areas of occupation which included the option of “other”. Comparable attention checks have been shown to improve the quality of data obtained through web-based surveys above that of classic paper and pencil questionnaires [57]. We asked participants to answer questions regarding protective behavior against COVID-19 (which we do not analyze here).

### Data analysis

#### Main analyses

Data analysis was carried out with hierarchical regression in R: models including each set of predictors and the control variables gender and age were compared to models in which the outcomes were regressed on the control variables. For all considered outcomes, the sets of predictors were the PID-5 scales (negative affectivity, detachment, antagonism, disinhibition, anankastia, and psychoticism) for Model 1, the motivational systems (BIS, FFFS, BAS-D, BAS- RR, and BAS-FS) for Model 2, and the IU scales (limited ability to act due to IU, burden due to IU, and vigilance due to IU) for Model 3. Variance inflation factors across the models never exceeded 3.2, meaning multicollinearity was not an issue. Values of continuous predictors were centered around their mean. Tables were computed using the package sjPlot in R. To complement the results of regression analyses with regard to the importance of predictors, we performed relative weight analyses (with package *rwa*, an implementation in R of [58]). Relative weights analysis permits obtaining more precise estimates of the relative importance of predictors compared with, for instance, beta coefficients from regression analysis [58].

#### Sensitivity analyses

We performed the regression analyses without the adjustment for control variables (see S1 Appendix).

Reporting for this manuscript was done in accordance with the STROBE [59] checklist.

## Results

Descriptive statistics and the correlations between the study variables are presented in Table A in the S1 Appendix, which also contains a brief comment on the Table. In what follows, we report on the results relating directly to our research questions.

### Depression and anxiety symptoms as outcomes

**Fig 1:**
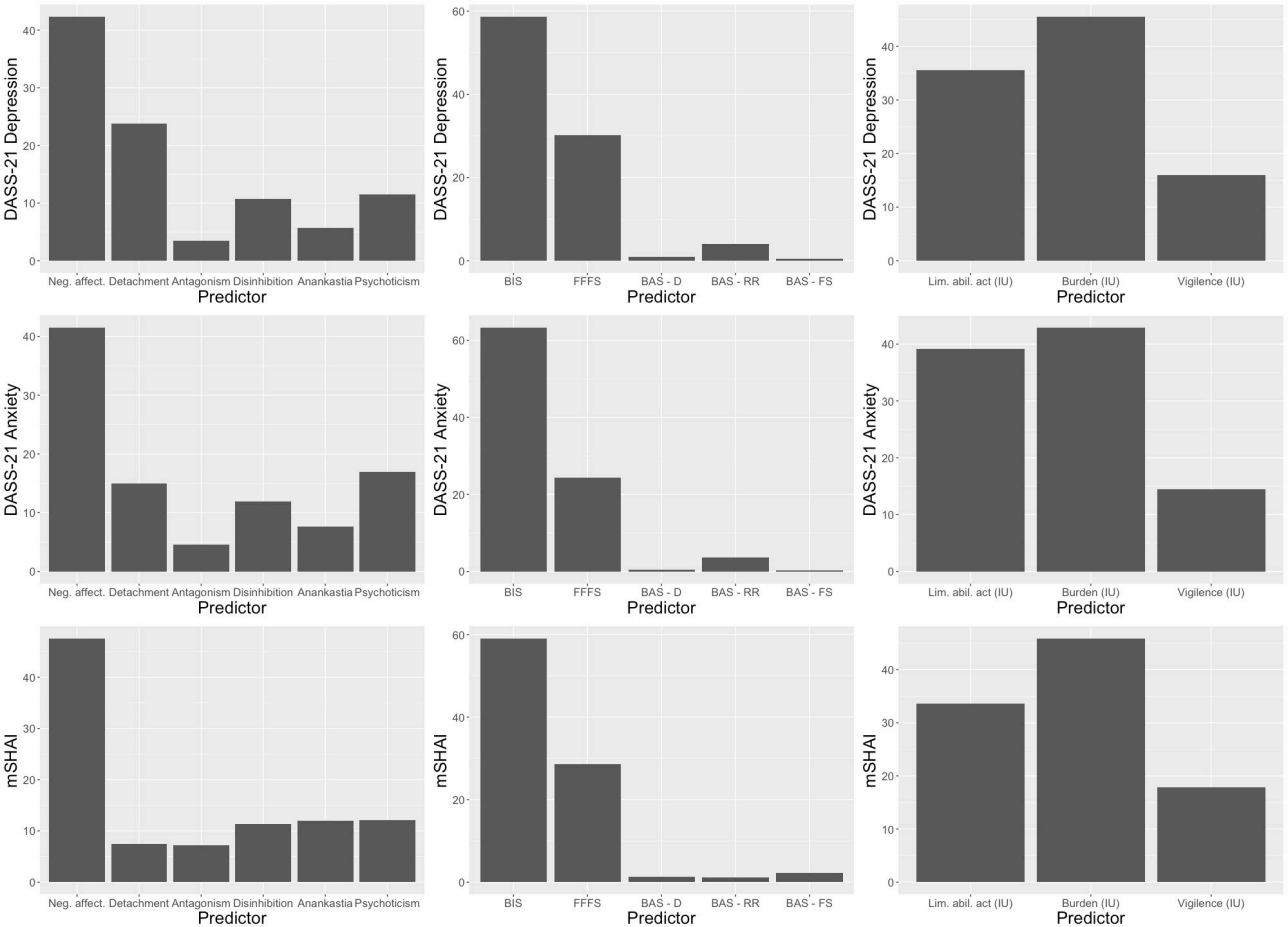
Relative weight analysis. The relative weights of maladaptive personality dimensions (column 1), motivational systems (column 2) and IU dimensions (column 3) in the prediction of depressive (row 1), anxious symptomatology (row 2) and health anxiety (row 3). Values indicate the percentage of the explained variance accounted for by each of the predictors. Relative weights of age and gender are not displayed here to enhance clarity.

The results of regression analyses with the depression and anxiety scales of the DASS-21 as outcomes are presented in Tables 2 and 3, respectively. The relative weights, indicating the importance of our predictors in the prediction of depression and anxiety symptoms, are indicated graphically in the first two rows of Fig 1.

**Table 2:** Regression analyses with depression as outcome.

			Depression			
	Model 1		Model 2		Model 3	
	*Beta*	95% C. I.	*Beta*	95% C. I.	*Beta*	95% C. I.
Intercept						
Gender	<0.01	-0.05 – 0.05	-0.08 *	-0.13 –-0.02	-0.01	-0.06 – 0.04
Age	-0.04	-0.09 – 0.01	-0.06 *	-0.12 – -0.01	-0.05 *	-0.10 – - <0.00
Negative affectivity	0.47 ***	0.41 – 0.53				
Detachment	0.30 ***	0.24 – 0.35				
Antagonism	-0.06	-0.12 – 0.01				
Disinhibition	<0.01	-0.06 – 0.07				
Anankastia	<0.01	-0.06 – 0.05				
Psychoticism	0.10 **	0.04 – 0.17				
Behavioral inhibition			0.40 ***	0.33 – 0.46		
Fight-Flight-Freeze			0.16 ***	0.09 – 0.23		
Drive			0.01	-0.06 – 0.08		
Reward responsiveness			-0.16 ***	-0.24 – -0.09		
Fun seeking			0.01	-0.05 – 0.08		
IU—Limit. act.					0.23 ***	0.15 – 0.31
IU—Burden					0.44 ***	0.36 – 0.53
IU—Vigilance					-0.02	-0.09 – 0.05
*R* ^*2*^	.47		.25		.40	
Delta *R*^*2*^	.45		.23		.38	
*F* Change	138.56 ***		60.468 ***		208.76 ***	

Note. 95% C. I.: 95% confidence interval. IU-Limit. Act: Limited ability to act due to intolerance of uncertainty. IU-Burden: Burden due to intolerance of uncertainty. IU-Vigilance: Vigilance due to intolerance of uncertainty. R^2^: coefficient of determination; Delta R^2^: Difference in the coefficient of determination in the comparison with a model including only the control variables as predictors; *F* Change: The F-value for the comparison between models.

*: *p* < .05;

**: *p* < .01;

***: *p* < .001.

**Table 3:** Regression analyses with anxiety as outcome.

			Anxiety			
	Model 1		Model 2		Model 3	
	*Beta*	95% C. I.	*Beta*	95% C. I.	*Beta*	95% C. I.
Intercept						
Gender	-0.01	-0.06 – 0.05	-0.07 *	-0.13 – -0.01	-0.03	-0.08 – 0.02
Age	-0.03	-0.09 – 0.02	-0.08 *	-0.13 – -0.02	-0.06 *	-0.11 – -0.01
Negative affectivity	0.42 ***	0.36 – 0.48				
Detachment	0.17 ***	0.11 – 0.23				
Antagonism	-0.04	-0.11 – 0.02				
Disinhibition	0.02	-0.05 – 0.09				
Anankastia	0.02	-0.03 – 0.08				
Psychoticism	0.19 ***	0.12 – 0.26				
Behavioral inhibition			0.38 ***	0.31 – 0.45		
Fight-Flight-Freeze			0.1 **	0.03 – 0.17		
Drive			0.03	-0.04 – 0.10		
Reward responsiveness			-0.15 ***	-0.23 – -0.08		
Fun seeking			0.03	-0.03 – 0.10		
IU—Limit. act.					0.28 ***	0.20 – 0.37
IU—Burden					0.38 ***	0.29 – 0.47
IU—Vigilance					-0.05	-0.12 – 0.02
*R* ^*2*^	.42		.19		.37	
Delta *R*^*2*^	.39		.17		.34	
*F* Change	111.79 ***		42.008 ***		178.97 ***	

Note. 95% C. I.: 95% confidence interval. IU-Limit. Act: Limited ability to act due to intolerance of uncertainty. IU-Burden: Burden due to intolerance of uncertainty. IU-Vigilance: Vigilance due to intolerance of uncertainty. R^2^: coefficient of determination; Delta R^2^: Difference in the coefficient of determination in the comparison with a model including only the control variables as predictors; *F* Change: The F-value for the comparison between models.

*: *p* < .05;

**: *p* < .01;

***: *p* < .001.

#### Depression symptoms

The delta *R*^*2*^ for Model 1 was .45 (additional 45% of variance explained by the maladaptive personality scales in comparison with the model including only the control variables). The highest beta value and relative weight was observed for negative affectivity and was followed by detachment. A similar pattern was observed in the relative weights (see Fig 1). Psychoticism was also a significant predictor. The motivational systems accounted for 23% of variance explained (Model 2). The BIS scale featured the highest beta and the highest relative weight. FFFS and BAS-RR were also associated with the depression scale (positively and negatively, respectively). The IU scales explained 38% of variance in the depression scale (Model 3). The scale relating to the burden due to IU featured the highest beta and the highest relative weight. The scale relating to the limited ability to act due to IU was also a significant predictor of the DASS-21 depression scale.

#### Anxiety symptoms

Maladaptive personality accounted for 39% of variance explained in the depression scale (Model 1). The highest beta value and relative weight were observed for negative affectivity, which was followed by psychoticism. Detachment was also a significant predictor. The delta *R*^*2*^ for Model 2 was .17. The BIS scale featured the highest beta, which was followed by the BAS-RR (negative association). The FFFS scale was also associated with the anxiety scale, which showed the second highest relative weights (here, the ranking of beta values and relative weight for the second highest value did not match). The IU scales accounted for 34% of variance explained in the anxiety subscale (Model 3). The scale relating to the burden due to IU featured the highest beta and relative weight. The limited ability to act due to IU scale (relative weight similar to burden due to IU) was also a significant predictor of the DASS-21 anxiety scale.

### Health anxiety symptoms as outcome

The results of regression analyses with health anxiety (mSHAI) as outcome are presented in Table 4, and Fig 1 (bottom row) depicts the relative weights. The maladaptive personality scales accounted for 35% of variance (Model 1). The highest beta value and relative weights were observed for negative affectivity. Anankastia and psychoticism were also significant predictors of mSHAI. The motivational systems scales explained 19% of variance in mSHAI (Model 2). The highest beta value and relative weight were observed for the BIS scale, which was followed by FFFS. BAS-D and BAS-FS were also positive predictors of mSHAI, and BAS-RR was negatively associated with mSHAI. Thirty percent of variance in mSHAI was explained by the IU scales (Model 3). The scale relating to the burden due to IU featured the highest beta and relative weight. The limited ability to act due to IU was also a significant predictor of mSHAI.

**Table 4:** Regression analyses with the health anxiety scales as outcome.

	Health anxiety					
	Model 1		Model 2		Model 3	
	*Beta*	95% C. I.	*Beta*	95% C. I.	*Beta*	95% C. I.
Intercept						
Gender	-0.09 **	-0.14 – -0.03	-0.17 ***	-0.23 – -0.11	-0.11 ***	-0.16 – -0.06
Age	0.08 **	0.03 – 0.13	0.05	-0.01 – 0.11	0.04	-0.02 – 0.09
Negative affectivity	0.46 ***	0.39 – 0.52				
Detachment	0.05	-0.01 – 0.11				
Antagonism	0.06	-0.01 – 0.12				
Disinhibition	0.02	-0.05 – 0.10				
Anankastia	0.09 **	0.03 – 0.15				
Psychoticism	0.09 *	0.02 – 0.17				
Behavioral inhibition			0.37 ***	0.30 – 0.44		
Fight-Flight-Freeze			0.17 ***	0.10 – 0.24		
Drive			0.07 *	0.00 – 0.15		
Reward responsiveness			-0.12 **	-0.19 – -0.04		
Fun seeking			0.09 **	0.03 – 0.16		
IU—Limit. act.					0.18 ***	0.09 – 0.26
IU—Burden					0.43***	0.33 – 0.52
IU—Vigilance					0.01	-0.07 – 0.08
*R* ^*2*^	.37		.22		.33	
Delta *R*^*2*^	.35		.19		.30	
*F* Change	97.69 ***		53.18 ***		159.78 ***	

Note. 95% C. I.: 95% confidence interval. IU-Limit. Act: Limited ability to act due to intolerance of uncertainty. IU-Burden: Burden due to intolerance of uncertainty. IU-Vigilance: Vigilance due to intolerance of uncertainty. R^2^: coefficient of determination; Delta R^2^: Difference in the coefficient of determination in the comparison with a model including only the control variables as predictors; *F* Change: The F-value for the comparison between models.

*: *p* < .05;

**: *p* < .01;

***: *p* < .001.

### Facets of fear of COVID-19 as outcomes

**Fig 2:**
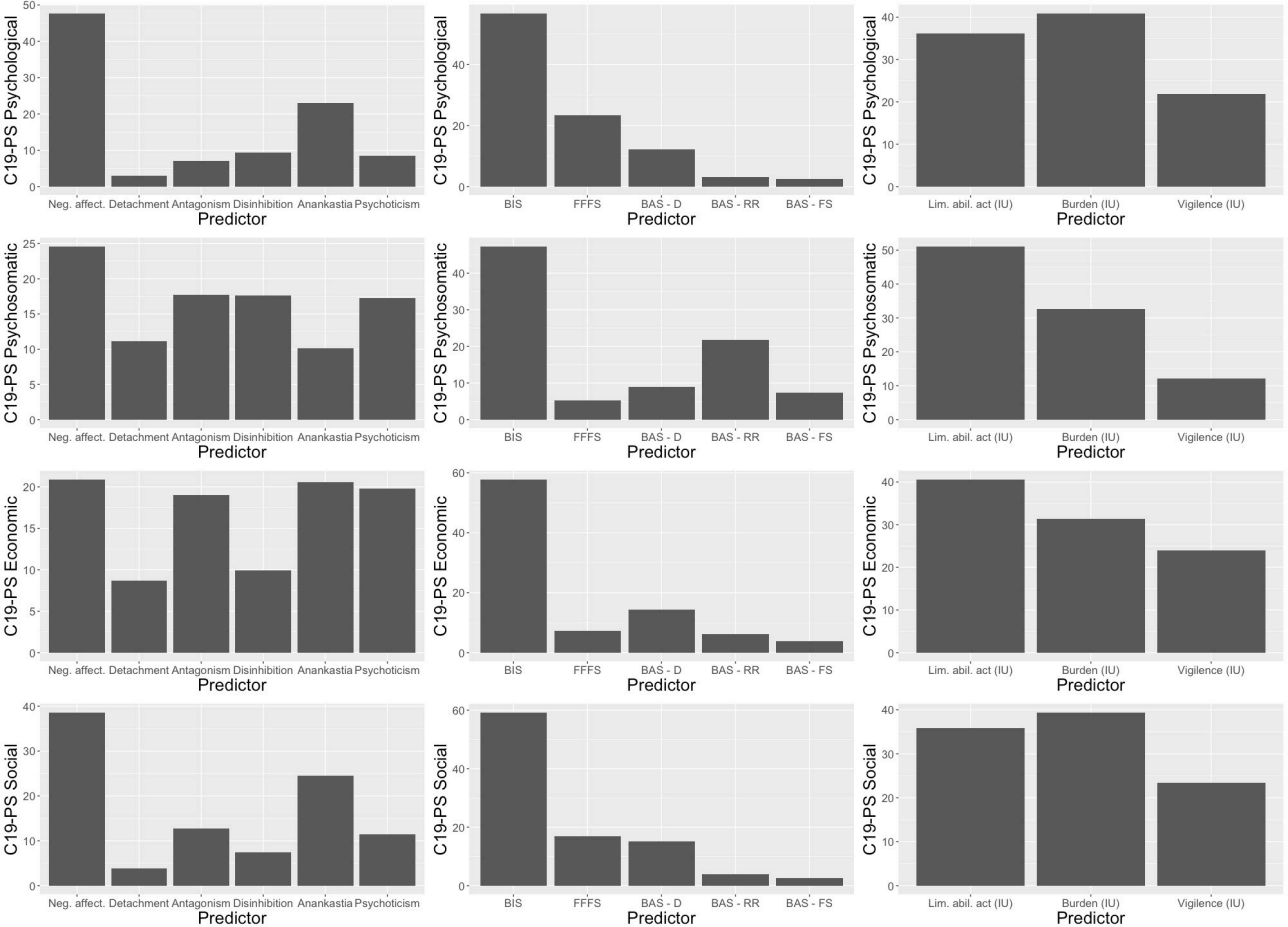
Relative weight analysis. The relative weights of maladaptive personality dimensions (column 1), motivational systems (column 2) and IU dimensions (column 3) in the prediction of COVID-19 Phobia scales: psychological (row 1), psychosomatic (row 2) and economic (row 3) and social (row 4) dimensions. Values indicate the percentage of the explained variance accounted for by each of the predictors. Relative weights of age and gender are not displayed here to enhance clarity.

Tables 5–8 present the results of regression analyses with the four included facets of fear of COVID-19 (four C19-PS scales) as outcomes. The relative weights, indicating the importance of our predictors in the prediction of each fear of COVID-19 factor are indicated graphically in Fig 2.

**Table 5:** Regression analyses with the psychological factor scale of the C19-PS instrument as outcome.

	Psychological					
	Model 1		Model 2		Model 3	
	*Beta*	95% C. I.	*Beta*	95% C. I.	*Beta*	95% C. I.
Intercept		-0.05 – 0.05		-0.06 – 0.06		-0.05 – 0.05
Gender	<0.01	-0.05 – 0.06	-0.06	-0.12 – 0.00	-0.01	-0.06 – 0.05
Age	0.03	-0.03 – 0.09	0.02	-0.04 – 0.08	>-0.01	-0.06 – 0.05
Negative affectivity	0.34 ***	0.27 – 0.41				
Detachment	-0.03	-0.10 – 0.04				
Antagonism	0.05	-0.02 – 0.13				
Disinhibition	0.03	-0.05 – 0.11				
Anankastia	0.17 ***	0.11 – 0.24				
Psychoticism	0.01	-0.07 – 0.10				
Behavioral inhibition			0.30 ***	0.23 – 0.38		
Fight-Flight-Freeze			0.11 **	0.04 – 0.19		
Drive			0.17 ***	0.09 – 0.24		
Reward responsiveness			-0.05	-0.13 – 0.02		
Fun seeking			0.03	-0.04 – 0.10		
IU—Limit. act.					0.19 ***	0.10 – 0.28
IU—Burden					0.27 ***	0.17 – 0.37
IU—Vigilance					0.06	-0.02 – 0.14
*R* ^*2*^	0.22		0.16		0.23	
Delta *R*^*2*^	0.20		0.13		0.21	
*F* Change	46.06 ***		35.43 ***		96.30 ***	

Note. 95% C. I.: 95% confidence interval. IU–Limit. Act: Limited ability to act due to intolerance of uncertainty. IU–Burden: Burden due to intolerance of uncertainty. IU- Vigilance: Vigilance due to intolerance of uncertainty. R^2^: coefficient of determination; Delta R^2^: Difference in the coefficient of determination in the comparison with a model including only the control variables as predictors; *F* Change: The F-value for the comparison between models.

*: *p* < .05;

**: *p* < .01;

***: *p* < .001.

**Table 6:** Regression analyses with the psycho-somatic factor scale of the C19-PS instrument as outcome.

	Psycho-somatic					
	Model 1		Model 2		Model 3	
	*Beta*	95% C. I.	*Beta*	95% C. I.	*Beta*	95% C. I.
Intercept		-0.05 – 0.05		-0.06 – 0.06		-0.06 – 0.06
Gender	0.02	-0.04 – 0.08	-0.03	-0.10 – 0.03	-0.04	-0.10 – 0.02
Age	0.01	-0.05 – 0.06	-0.06	-0.12 – 0.01	-0.06 *	-0.12 – -0.00
Negative affectivity	0.19 ***	0.12 – 0.26				
Detachment	0.07 *	0.00 – 0.13				
Antagonism	0.14 ***	0.06 – 0.21				
Disinhibition	0.09 *	0.01 – 0.17				
Anankastia	0.06	-0.01 – 0.12				
Psychoticism	0.10 *	0.02 – 0.18				
Behavioral inhibition			0.27 ***	0.20 – 0.35		
Fight-Flight-Freeze			-0.09 *	-0.17 – -0.01		
Drive			0.12 **	0.05 – 0.20		
Reward responsiveness			-0.25 ***	-0.33 – -0.17		
Fun seeking			0.10 **	0.02 – 0.17		
IU—Limit. act.					0.32 ***	0.22 – 0.41
IU—Burden					0.13 *	0.03 – 0.24
IU—Vigilance					-0.06	-0.14 – 0.02
*R* ^*2*^	0.24		0.08		0.16	
Delta *R*^*2*^	0.21		0.06		0.14	
*F* Change	48.57 ***		15.08 ***		59.68 ***	

Note. 95% C. I.: 95% confidence interval. IU–Limit. Act: Limited ability to act due to intolerance of uncertainty. IU–Burden: Burden due to intolerance of uncertainty. IU- Vigilance: Vigilance due to intolerance of uncertainty. R^2^: coefficient of determination; Delta R^2^: Difference in the coefficient of determination in the comparison with a model including only the control variables as predictors; *F* Change: The F-value for the comparison between models.

*: *p* < .05;

**: *p* < .01;

***: *p* < .001.

**Table 7:** Regression analyses with the economic factor scale of the C19-PS instrument as outcome.

	Economic					
	Model 1		Model 2		Model 3	
	*Beta*	95% C. I.	*Beta*	95% C. I.	*Beta*	95% C. I.
Intercept		-0.05 – 0.05		-0.06 – 0.06		-0.06 – 0.06
Gender	-0.01	-0.07 – 0.05	-0.07 *	-0.13 – -0.00	-0.06 *	-0.12 – -0.00
Age	0.02	-0.04 – 0.08	-0.04	-0.10 – 0.02	-0.04	-0.10 – 0.02
Negative affectivity	0.17 ***	0.10 – 0.24				
Detachment	0.04	-0.03 – 0.11				
Antagonism	0.15 ***	0.07 – 0.22				
Disinhibition	<0.01	-0.08 – 0.08				
Anankastia	0.14 ***	0.07 – 0.20				
Psychoticism	0.13 **	0.05 – 0.22				
Behavioral inhibition			0.26 ***	0.19 – 0.34		
Fight-Flight-Freeze			-0.1 *	-0.17 – -0.02		
Drive			0.13 **	0.05 – 0.21		
Reward responsiveness			-0.14 **	-0.23 – -0.06		
Fun seeking			0.04	-0.03 – 0.12		
IU—Limit. act.					0.21 ***	0.12 – 0.31
IU—Burden					0.12 *	0.01 – 0.22
IU—Vigilance					0.08	-0.00 – 0.16
*R* ^*2*^	0.22		0.06		0.14	
Delta *R*^*2*^	0.20		0.04		0.12	
*F* Change	45.08 ***		11.87 ***		52.65 ***	

Note. 95% C. I.: 95% confidence interval. IU-Limit. Act: Limited ability to act due to intolerance of uncertainty. IU-Burden: Burden due to intolerance of uncertainty. IU-Vigilance: Vigilance due to intolerance of uncertainty. R^2^: coefficient of determination; Delta R^2^: Difference in the coefficient of determination in the comparison with a model including only the control variables as predictors; *F* Change: The F-value for the comparison between models.

*: *p* < .05;

**: *p* < .01;

***: *p* < .001.

**Table 8:** Regression analyses with the social factor scale of the C19-PS instrument as outcome.

	Social					
	Model 1		Model 2		Model 3	
	*Beta*	95% C. I.	*Beta*	95% C. I.	*Beta*	95% C. I.
Intercept		-0.06 – 0.06		-0.06 – 0.06		-0.06 – 0.06
Gender	0.02	-0.04 – 0.08	-0.05	-0.11 – 0.01	-0.01	-0.07 – 0.05
Age	0.04	-0.02 – 0.09	0.01	-0.05 – 0.07	-0.01	-0.07 – 0.05
Negative affectivity	0.28 ***	0.21 – 0.35				
Detachment	-0.01	-0.08 – 0.05				
Antagonism	0.12 **	0.04 – 0.20				
Disinhibition	-0.02	-0.11 – 0.06				
Anankastia	0.16 ***	0.09 – 0.23				
Psychoticism	0.05	-0.04 – 0.13				
Behavioral inhibition			0.29 ***	0.21 – 0.36		
Fight-Flight-Freeze			0.06	-0.02 – 0.13		
Drive			0.16 ***	0.08 – 0.24		
Reward responsiveness			-0.05	-0.13 – 0.03		
Fun seeking			0.03	-0.05 – 0.10		
IU—Limit. act.					0.16 **	0.07 – 0.26
IU—Burden					0.22 ***	0.12 – 0.32
IU—Vigilance					0.07	-0.01 – 0.15
*R* ^*2*^	0.20		0.12		0.17	
Delta *R*^*2*^	0.18		0.10		0.15	
*F* Change	40.38 ***		25.95 ***		67.89 ***	

Note. 95% C. I.: 95% confidence interval. IU-Limit. Act: Limited ability to act due to intolerance of uncertainty. IU-Burden: Burden due to intolerance of uncertainty. IU-Vigilance: Vigilance due to intolerance of uncertainty. R^2^: coefficient of determination; Delta R^2^: Difference in the coefficient of determination in the comparison with a model including only the control variables as predictors; *F* Change: The F-value for the comparison between models.

*: *p* < .05;

**: *p* < .01;

***: *p* < .001.

#### Psychological factor

Maladaptive personality scales explained 20% of variance in the psychological factor (Model 1, Table 5). The highest beta value and relative weight were observed for negative affectivity. Anankastia was also a significant predictor of the psychological factor. Thirteen percent of variance were explained by the motivational systems scales (Model 2). The BIS scale featured the highest beta value and relative weight. The FFFS and BAS–D scales were positive predictors of the psychological factor. The IU scales accounted for 21% of variance in the psychological factor. The scale relating to the burden due to IU featured the highest beta value and relative weight. The limited ability to act due to IU scale was also a significant predictor of the psychological factor.

#### Psycho-somatic factor

Maladaptive personality scales accounted for 21% of variance in the psycho-somatic factor (Model 1, Table 6). The highest beta value and relative weight were observed for negative affectivity, which was followed in the case of the beta weight by antagonism. Detachment, disinhibition, and psychoticism were also significant predictors of the psycho-somatic factor. All three featured similar relative weights. Only 6% of variance in psycho-somatic difficulties were explained by the motivational systems (Model 2). The BIS scale featured the highest beta value and relative weights, which was closely followed by the BAS-RR scale (negative association). The FFFS scale was also a negative predictor of the psycho-somatic factor, while a positive association with psycho-somatic difficulties was observed for the BAS-D and BAS-FS scales. The IU scales accounted for 14% of variance in psycho-somatic factor scores (Model 3). The scale relating to the limited ability to act due to IU featured the highest beta value and relative weight. The burden due to IU scale was also a significant predictor of the psycho-somatic factor.

#### Economic factor

Twenty percent of variance in economic difficulties were explained by the maladaptive personality scales (Model 1, Table 7). Again, the highest beta value and relative weights were observed for negative affectivity, which was followed by antagonism in the case of the beta value. Yet, relative weights showed a similar contribution of negative affectivity, antagonism, anankastia, and psychoticism, all of which also were significant predictors of the economic factor. The motivational systems accounted for only 4% of variance in economic difficulties scores (Model 2). The highest beta and relative weight were observed for the BIS, which was followed by the BAS-RR scale (negative association). The FFFS scale was also a negative predictor of the economic factor, while a positive association with economic difficulties was observed for the BAS-D and BAS-FS scales. The IU scales explained 12% of variance in the economic factor (Model 3). The scale relating to the limited ability to act due to IU featured the highest beta value and relative weight. The burden due to IU scale was also a significant predictor of the economic factor.

#### Social factor

The maladaptive personality scales explained 18% of variance in social difficulties scores (Model 1, Table 8). The highest beta value and relative weight were observed for negative affectivity, which was followed by anankastia. Antagonism was also a significant predictor of the social factor. The motivational systems accounted for only 10% of variance in social difficulties scores (Model 2). The BIS scale featured the highest beta value. The BAS-D scale was also a predictor of the social factor. Fifteen percent of variance in social difficulties scores were explained by the IU scales (Model 3). The burden due to IU scale featured the highest beta value and relative weight. The scale related to the limited ability to act due to IU was also a significant predictor of the social factor.

### Similarities in predictor groups and predictors within groups between outcomes

The models including maladaptive personality dimensions as predictors explained 45% of symptoms of depression and 39% of symptoms of anxiety over the effect of gender and age (delta *R*^*2*^). This was similar to the models including IU scales as predictors which explained 38% and 34% of variance in these constructs, respectively, again beyond the effect of gender and age. In comparison, the motivational systems explained between one half to two thirds of the variance accounted for by the mentioned constructs in the outcomes (23% and 17%, respectively).

A portion of 35% of variance in the more specific outcome health anxiety was explained by the maladaptive personality dimensions (again, above the effect of gender and age). The IU scales accounted for 30% and the motivational systems for 19% of the variance in health anxiety. The portion of variance explained in the even more specific COVID-19 Phobia scales was substantially smaller. Note the predicted variance for each outcome amounts to more than 100% because different models were built for each set of predictors.

Turning to similarities in predictors within groups, negative affectivity was an important predictor regardless of the mental health outcomes. Psychoticism was associated with anxiety, and depression and health anxiety symptoms were associated with two of the fear of COVID-19 factors (psycho-somatic and economic). Detachment was an important predictor of depression symptoms and was significantly associated with anxiety symptoms as well as psychosomatic symptoms (fear of COVID-19). Antagonism was not significantly associated with either depression or anxiety symptoms, nor with health anxiety, but it was a significant predictor of three of the fear of COVID-19 factors (psycho-somatic, economic, and social). Anankastia was associated with health anxiety and was an important predictor of three of the fear of COVID-19 factors (but not of the psycho-somatic dimension: non-significant association).

Burden due to IU scale was an important predictor of depression and anxiety as well as of health anxiety, and the psychological and social dimensions of fear of COVID-19. It was also significantly associated with the psychosomatic and economic dimensions. The limited ability to act due to IU scale was significantly associated with depression, anxiety, and health anxiety. It was significantly associated with all of the fear of COVID-19 factors (important predictor of psychosomatic economic factors). However, in our regression models, vigilance due to IU was not associated with any of the outcomes.

The BIS was an important predictor of all of the outcomes, and the FFFS was significantly associated with each outcome except for the social dimension of fear of COVID-19. The association was negative for the psychosomatic and economic dimensions. The BAS-D scale was significantly associated with symptoms of health anxiety as well as with all of the dimensions of fear of COVID-19. Contrary to expectations, these associations were positive. As expected, BAS-RR was negatively associated with all of the outcomes, with the exception of the psychological and social dimensions of fear of COVID-19. Finally, BAS-FS was not associated with depression and anxiety, but it was positively associated with health anxiety (positively, contrary to expectations) and negatively associated with the psychosomatic dimension of fear of COVID-19.

#### Sensitivity analyses

We compared these results with those of unadjusted analyses (see Tables B to H in the S1 Appendix). In two cases, the effect was non-significant in the adjusted model but significant in the non-adjusted model (mSHAI regressed on detachment in the Maladaptive personality models, and the economic factor of fear of COVID-19 regressed on the IU-Vigilance in the IU model). The opposite was observed for mSHAI regressed on BAS-RR in the motivational systems model. For all other results, the same conclusions were reached in adjusted and non-adjusted models. In almost all instances, the estimates either did not differ between adjusted and non-adjusted models, or they differed by one unit at the second decimal only.

## Discussion

Beyond a conceptual replication of past research findings, a main contribution of this study is the comparison of the dimensions of maladaptive personality, the motivational systems and IU in the prediction of symptoms of emotional disorders (anxiety and depression) and more specific symptoms, such as those of health anxiety and fear of COVID-19. This is important because past research examining the association of maladaptive personality, the motivational systems (Gray’s theory), and IU has been highly compartmentalized. Thus, which group of predictors shares the most variance with our studied outcomes remained an important research question in need of an answer, which we have endeavoured to provide here. Further aims of this study were to identify, in each group of constructs, the significant predictors beyond the effect of others and to examine regularities in order to determine their importance in the prediction of our outcome variables. Our results have shown that in relation to depression and anxiety, a relatively large (34% to 45%) variance in these general outcomes was shared with maladaptive personality and IU, whereas the more specific outcomes shared less variance with these: 19% to 35% for health anxiety and 4% to 21% for fear of COVID-19, depending on the subscale. The variance that depression and (health) anxiety shared with maladaptive personality predictors was overall about twice the variance they shared with this group of predictors; and IU shared 72% more variance with depression and (health) anxiety than the BIS/BAS/FFFS predictor group did. On average, the variance shared between fear of COVID-19 and maladaptive personality was three times the variance these outcomes shared with BIS/BAS/FFFS, whilst IU shared twice as much variance with these outcomes as BIS/BAS/FFFS did. Thus, all in all, our study shows that maladaptive personality and IU dimensions are stronger sets of predictors of the studied emotional symptoms than are the motivational system dimensions. The relatively strong focus of past research on the BIS/BAS and FFFS as prodromes of psychopathology [22] might be due more to the advantage of Gray’s theory in terms of theoretical (see Introduction) [27] rather than statistical explainability of mental health outcomes.

Among the predictor groups, the relative weights analysis portion of our study demonstrates the relative importance of negative affectivity (maladaptive personality dimension), the BIS (motivational systems dimension), and limited ability to act due to IU (IU dimension) in particular. The relative importance of other predictors was generally lower and varied more between outcomes. The importance of negative affectivity in mental health was already highlighted in previous research focusing on depression and anxiety (e.g., [1, 21]) as well as on COVID-19 related anxiety [10]. Our regression models and relative weights analyses show that detachment and psychoticism might play a lesser role in this study than in previous ones (e.g., [1, 10]). Past research also highlighted the respective roles of the BIS (e.g., [2, 29, 30]) and IU (e.g., [40]) in mental health outcomes and found IU to be related to anxiety with regard to COVID-19 ([4, 44]). This had not as yet been tested in a German sample.

In our study, we examined psychopathology by relying upon a dimensional perspective, which is more in line with recent conceptualizations of mental health (such as the Research Domain Criteria; [60]) which emphasize the “[c]ontinuity between normative personality and pathological personality” [61]. A dimensional approach–rather than considering psychopathology and normality in terms of polar opposites–allowed us to examine the associations between our variables of interest conceptualized in terms of degree (focusing on symptoms rather than disorders), which seems a more valid approach [62]. Indeed, most mental health disorders have no natural boundaries that distinguish them from the normal or normative (absence of a zone of rarity; [63]), and thus the question of validity and of reliability has always been a conundrum of the categorical approach to mental health [60, 61, 63]. Further, investigating symptomatology rather than full-blown disorders is relevant on account of the impact of subthreshold depression and anxiety (e.g., [46, 64, 65]).

### Interpretation of our findings

Our findings show that dispositional factors share an important part of variance in depression, anxiety, health anxiety, and a smaller yet significant part of variance with the fear of COVID-19 factors. The interpretation of our results should take into account the number of tests that were carried out. It can be expected that 5% of tests are significant in the absence of a real effect. In our study, all the regression models showed significance above the control models at *p* < .001, and around two out of three of the individual regression coefficients reached significance (more than half of the total had *p* values inferior to .001), but it is possible that a few of these associations might not hold in another sample (e.g., among the minority with a *p* value that was not inferior to .001); for example, we mentioned above that, in two cases, the effect was non-significant in the adjusted model but significant in the non-adjusted model.

### Implications

Our findings also have research implications with regard to the variables to be assessed as a matter of priority in mental health research, which is often constrained by financial costs and seeks to avoid unnecessary participant burden. The question of the prioritization of variables is even more relevant for longitudinal studies. For instance, relying upon information provided by both the *R2* values and the relative weight, negative affectivity, and burden due to IU overall each account for around 40% more variance in depression and anxiety symptoms than the BIS, above and beyond the variance explained by other variables included in the respective models. This relative difference is of yet more significance for health anxiety (about 75% and 55% more variance explained by negative affectivity and burden due to IU compared with the BIS). We note the pattern can sometimes be distinguished between outcomes. For instance, negative affectivity, the BIS, and burden due to IU each account for 9% to 10% of variance in the psychological factor (fear of COVID-19) above and beyond other variables included in our models. We suggest these aspects should be examined further in longitudinal studies to clarify the direction of the effects we documented, and whether the similarity between outcomes remains in longitudinal models.

Our findings have implications for clinical practice as well, as they highlight the dimensions to work on during psychotherapy: the dimensions of the dispositional factor groups which are strongly associated with all outcomes. Indeed, unlike genes, dispositional traits can be changed, notably through psychotherapy [66, 67] within the therapeutic range ([68–70]). Promising approaches to lasting personality change are the volitional performance by the client of behavior congruent with the desired traits combined with congruent experiences, as well as mindfulness meditation [65], i.e., training voluntary focused attention and non-judgmental acceptance, notably [71, 72].

As also observed in previous research (e.g., [10, 70]), our findings underline the importance of negative affectivity in the context of depressive and anxious symptomatology, as well as health anxiety and fear of COVID-19. Negative affectivity (neuroticism) is fortunately the personality dimension that is the most responsive to psychotherapeutic intervention [69]. Focusing psychotherapy on reducing limited ability to act due to IU and possibly to a lesser extent also on the BIS might act synergically with reductions in negative affectivity in terms of alleviating patients’ depressive and anxious symptomatology.

### Limitations

The recruitment of participants for our study was limited to Germany. This is an advantage from the perspective of transcultural research as there is not much dimensional research with German participants, and our study has shown that associations expected based on research findings mainly from the USA were found in Germany as well. Yet, recruitment of participants in a single country potentially limits generalizability (see below). This study is a cross-sectional study, and it is thus not possible for us to exclude reverse relationships such as changes in personality stemming from fear of COVID-19, for instance. The dimensional factors we used as predictors are theoretically more stable than our outcome variables, and we therefore assume that reverse associations would be of a smaller magnitude.

### Generalizability

Given the homogeneous nature of the non-clinical sample population we recruited for this cross-sectional study (exclusively in Germany), our results might not generalize to other countries or to clinical samples.

## Conclusion

Our study has demonstrated the close similarity between symptoms of depression and anxiety in terms of the importance of the different sets of dispositional dimensions used as predictors, as well as the contribution of these sets of dispositional dimensions to both outcomes in terms of the variance predicted.

## Supporting information

S1 Appendix Supplementary appendix to the manuscript titled dispositional factors in the explanation of symptoms of depression, anxiety, health anxiety and COVID-19 Phobia. (DOCX)
