# Supplementary material for: Dispositional factors in the explanation of symptoms of depression, anxiety, health anxiety and COVID-19 Phobia
Source: PLoS One. 2024 Apr 16;19(4):e0299593. doi: 10.1371/journal.pone.0299593 (PMC11020815; doi:10.1371/journal.pone.0299593)
Supplement: S1 Appendix — (DOCX) [file pone.0299593.s001.docx]

Supplementary appendix to the manuscript titled

Dispositional factors in the explanation of symptoms of depression, anxiety, health anxiety and COVID-19 Phobia

We limit our comments of the correlations between our study variables to the associations within and between sets of predictor constructs. Small to medium correlations were observed among the maladaptive personality dimensions (average *r* = .45). The dimension with the largest associations on average was psychoticism, ranging from *r* = .47 (with negative affectivity) to .62 (with disinhibition). In addition, the correlation of disinhibition with anankastia was .27, other non-mentioned correlations with disinhibition ranged from .49 (with detachment) to .56 (with antagonism). Finally, the rest of the associations between maladaptive personality dimensions ranged from .35 to .42.

Among the motivational systems set of constructs, the FFFS was moderately correlated with the BIS (*r* = .58). Other correlations with the FFFS were very small (*r*s ranging from -.11 to .02). In addition, the correlation of the BIS with BAS – RR was small (*r* = .22), and the correlation with the BAS – FS was small (*r* = .064). Correlations of medium size were observed within the BAS dimensions, ranging from .47 to .61. The largest correlations were observed among the IU dimensions (*r*s ranging from .64 to .69, meaning 41 to 47% of the variance was shared between the dimensions).

Among the associations of dimensions of different sets of predictors, negative affectivity and the BIS (*r =* .59) and FFFS (*r =* .48). Other associations between maladaptive personality dimensions and the motivational systems were (very) small or non-significant. Moderate correlations were also observed between negative affectivity with Burden due to IU (*r* = .66) and Limited ability to act due to IU (*r* = .58). Anankastia was moderately associated with Vigilance due to IU (*r* = .50). Other correlations between maladaptive personality dimensions and IU scales ranged from .24 to .41. The BIS was moderately associated with the IU scales (correlations ranging from .44 for Vigilance due to IU to .64 for Burden due to IU). The FFFS was moderately associated with Burden due to IU (*r* = .54) and the FFFS with Limited ability to act due to IU (*r* = .43). The associations between the BAS scales and Limited ability to act due to IU and Burden due to IU ranged from non-significant to very small. Vigilance due to IU was non significantly associated with the BAS – FS. Other associations between the motivational systems and the IU scales were small (*r*s ranging from .18 to .28).

Table A. Descriptive statistics and correlations among the study variables (continues)

|  | Mean | SD | 1 | 2 | 3 | 4 | 5 | 6 | 7 | 8 | 9 | 10 | 11 | 12 | 13 |
| --- | --- | --- | --- | --- | --- | --- | --- | --- | --- | --- | --- | --- | --- | --- | --- |
| 1. Gender | 0.50 | 0.50 | - |  |  |  |  |  |  |  |  |  |  |  |  |
| 2. Age | 47.26 | 13.94 | -0.19 | - |  |  |  |  |  |  |  |  |  |  |  |
| 3. Negative affectivity | 1.08 | 0.64 | 0.17 | -0.20 | (.82) |  |  |  |  |  |  |  |  |  |  |
| 4. Detachment | 0.88 | 0.54 | -0.08 | -0.04 | 0.35 | (.75) |  |  |  |  |  |  |  |  |  |
| 5. Antagonism | 0.70 | 0.56 | -0.17 | -0.23 | 0.35 | 0.42 | (.79) |  |  |  |  |  |  |  |  |
| 6. Disinhibition | 0.75 | 0.53 | -0.02 | -0.16 | 0.54 | 0.49 | 0.56 | (.77) |  |  |  |  |  |  |  |
| 7. Anankastia | 1.17 | 0.61 | -0.05 | -0.09 | 0.41 | 0.35 | 0.41 | 0.27 | (.81) |  |  |  |  |  |  |
| 8. Psychoticism | 0.86 | 0.58 | -0.06 | -0.17 | 0.47 | 0.52 | 0.59 | 0.62 | 0.49 | (.79) |  |  |  |  |  |
| 9. Behavioral inhibition | 2.75 | 0.71 | 0.23 | -0.19 | 0.59 | 0.22 | 0.12 | 0.32 | 0.26 | 0.25 | (.81) |  |  |  |  |
| 10. Fight-Flight-Freeze | 2.83 | 0.69 | 0.28 | -0.16 | 0.48 | 0.14 | -0.06 | 0.19 | 0.07 | 0.05 | 0.58 | (.72) |  |  |  |
| 11. BAS - Drive | 2.75 | 0.55 | 0.01 | -0.14 | 0.05 | -0.15 | 0.20 | -0.02 | 0.32 | 0.11 | 0.09 | -0.11 | (.71) |  |  |
| 12. BAS - Reward responsiveness | 3.00 | 0.49 | 0.02 | -0.05 | 0.09 | -0.24 | 0.07 | <0.01 | 0.17 | 0.08 | 0.22 | 0.02 | 0.61 | (.67) |  |
| 13. BAS - Fun seeking | 2.66 | 0.56 | <0.01 | -0.14 | 0.06 | -0.11 | 0.23 | 0.26 | 0.08 | 0.24 | 0.06 | -0.09 | 0.47 | 0.51 | (.66) |
| 14. IU - Lim. Act. | 2.56 | 0.97 | 0.06 | -0.12 | 0.58 | 0.41 | 0.30 | 0.43 | 0.35 | 0.39 | 0.59 | 0.43 | 0.03 | 0.07 | 0.01 |
| 15. IU - Stress | 2.84 | 1.01 | 0.14 | -0.17 | 0.66 | 0.37 | 0.25 | 0.38 | 0.40 | 0.37 | 0.64 | 0.54 | 0.07 | 0.11 | -0.01 |
| 16. IU - Vigilance | 3.02 | 0.81 | -0.03 | -0.09 | 0.44 | 0.34 | 0.28 | 0.24 | 0.50 | 0.33 | 0.44 | 0.28 | 0.21 | 0.18 | 0.01 |
| 17. DASS-21 - Depression | 0.74 | 0.78 | 0.07 | -0.15 | 0.61 | 0.49 | 0.30 | 0.44 | 0.32 | 0.45 | 0.45 | 0.38 | -0.06 | -0.06 | -0.05 |
| 18. DASS-21 - Anxiety | 0.56 | 0.65 | 0.06 | -0.15 | 0.58 | 0.42 | 0.32 | 0.44 | 0.34 | 0.48 | 0.40 | 0.30 | -0.02 | -0.03 | -0.01 |
| 19. DASS-21 - Stress | 0.87 | 0.77 | 0.14 | -0.17 | 0.64 | 0.41 | 0.31 | 0.49 | 0.37 | 0.47 | 0.50 | 0.43 | 0.04 | 0.04 | 0.02 |
| 20. mSHAI | 1.16 | 0.94 | -0.05 | -0.04 | 0.56 | 0.33 | 0.34 | 0.40 | 0.37 | 0.42 | 0.41 | 0.31 | 0.05 | 0.06 | 0.07 |
| 21. C19PS - Psyological | 2.24 | 1.01 | 0.04 | -0.07 | 0.44 | 0.19 | 0.25 | 0.28 | 0.33 | 0.29 | 0.36 | 0.25 | 0.16 | 0.13 | 0.09 |
| 22. C19PS - Somatic | 1.37 | 0.69 | 0.01 | -0.11 | 0.39 | 0.31 | 0.36 | 0.38 | 0.29 | 0.39 | 0.18 | 0.04 | 0.06 | -0.06 | 0.06 |
| 23. C19PS - Economic | 1.50 | 0.79 | -0.03 | -0.08 | 0.35 | 0.28 | 0.36 | 0.31 | 0.34 | 0.39 | 0.18 | 0.02 | 0.10 | 0.01 | 0.06 |
| 24. C19PS - Social | 1.91 | 0.97 | 0.03 | -0.07 | 0.39 | 0.20 | 0.28 | 0.26 | 0.33 | 0.30 | 0.31 | 0.19 | 0.16 | 0.13 | 0.09 |

Note. BIS: Behavioral inhibition system (Heym et al., 2020); FFFS: Fight-Flight-Freeze (Heym et al., 2008); BAS: Behavioral Activation System; IU – Lim. act.: Limited ability to act due to Intolerance to uncertainty; IU - Stress: Stress due to Intolerance of uncertainty; IU - Vigilance: Vigilance related to intolerance of uncertainty – Vigilance. mSHAI: modified Short Health Anxiety Inventory; C19PS: Covid-19 phobia scale. Correlations with values higher than 0.06 are significant at p < .05.

Table A. Descriptive statistics and correlations among the study variables (continued)

|  | 14 | 15 | 16 | 17 | 18 | 19 | 20 | 21 | 22 | 23 | 24 |
| --- | --- | --- | --- | --- | --- | --- | --- | --- | --- | --- | --- |
| 13. BAS - Fun seeking |  |  |  |  |  |  |  |  |  |  |  |
| 14. IU - Lim. Act. | (.89) |  |  |  |  |  |  |  |  |  |  |
| 15. IU - Stress | 0.79 | (.91) |  |  |  |  |  |  |  |  |  |
| 16. IU - Vigilance | 0.64 | 0.68 | (.81) |  |  |  |  |  |  |  |  |
| 17. DASS-21 - Depression | 0.57 | 0.62 | 0.43 | (.93) |  |  |  |  |  |  |  |
| 18. DASS-21 - Anxiety | 0.56 | 0.58 | 0.40 | 0.79 | (.89) |  |  |  |  |  |  |
| 19. DASS-21 - Stress | 0.57 | 0.65 | 0.46 | 0.83 | 0.78 | (.91) |  |  |  |  |  |
| 20. mSHAI | 0.50 | 0.55 | 0.41 | 0.51 | 0.58 | 0.53 | (.96) |  |  |  |  |
| 21. C19PS - Psyological | 0.44 | 0.46 | 0.37 | 0.37 | 0.45 | 0.41 | 0.54 | (.90) |  |  |  |
| 22. C19PS - Somatic | 0.39 | 0.35 | 0.24 | 0.40 | 0.53 | 0.39 | 0.50 | 0.60 | (.93) |  |  |
| 23. C19PS - Economic | 0.36 | 0.34 | 0.30 | 0.36 | 0.46 | 0.35 | 0.50 | 0.55 | 0.74 | (.89) |  |
| 24. C19PS - Social | 0.38 | 0.40 | 0.33 | 0.34 | 0.43 | 0.38 | 0.54 | 0.75 | 0.65 | 0.66 | (.89) |

Note. BIS: Behavioral inhibition system (Heym et al., 2020); FFFS: Fight-Flight-Freeze (Heym et al., 2008); BAS: Behavioral Activation System; IU – Lim. act.: Limited ability to act due to Intolerance to uncertainty; IU - Stress: Stress due to Intolerance of uncertainty; IU - Vigilance: Vigilance related to intolerance of uncertainty – Vigilance. mSHAI: modified Short Health Anxiety Inventory; C19PS: Covid-19 phobia scale. Correlations with values higher than 0.06 are significant at *p* < .05.

Table B. Regression analyses with the Depression as outcome

|  |  |  | Depression |  |  |  |
| --- | --- | --- | --- | --- | --- | --- |
|  | Model 1 | | Model 2 | | Model 3 | |
|  | *Beta* | 95% C. I. | *Beta* | 95% C. I. | *Beta* | 95% C. I. |
| Intercept |  |  |  |  |  |  |
| Negative affectivity | 0.47 *** | 0.42 – 0.53 |  |  |  |  |
| Detachment | 0.29 *** | 0.24 – 0.35 |  |  |  |  |
| Antagonism | -0.05 | -0.11 – 0.01 |  |  |  |  |
| Disinhibition | <0.001 | -0.06 – 0.07 |  |  |  |  |
| Anankastia | -0.01 | -0.06 – 0.05 |  |  |  |  |
| Psychoticism | 0.11 ** | 0.04 – 0.18 |  |  |  |  |
| Behavioral inhibition |  |  | 0.40 *** | 0.33 – 0.47 |  |  |
| Fight-Flight-Freeze |  |  | 0.15 *** | 0.08 – 0.22 |  |  |
| Drive |  |  | 0.02 | -0.06 – 0.09 |  |  |
| Reward responsiveness |  |  | -0.17 *** | -0.24 – -0.10 |  |  |
| Fun seeking |  |  | 0.02 | -0.04 – 0.09 |  |  |
| IU - Limit. act. |  |  |  |  | 0.23 *** | 0.15 – 0.31 |
| IU - Burden |  |  |  |  | 0.45 *** | 0.37 – 0.53 |
| IU - Vigilance |  |  |  |  | -0.02 | -0.09 – 0.05 |
| *R^2^* | .47 |  | .24 |  | .40 |  |

Note. 95% C. I.: 95% confidence interval. IU – Limit. Act: Limited ability to act due to intolerance of uncertainty. IU – Burden: Burden due to intolerance of uncertainty. IU- Vigilance: Vigilance due to intolerance of uncertainty. R^2^: coefficient of determination; Delta R^2^: Difference in the coefficient of determination in the comparison with a model including only the control variables as predictors; *: *p* < .05; **: *p* < .01; ***: *p* < .001.

Table C. Regression analyses with Anxiety as outcome

|  |  |  | Anxiety |  |  |  |
| --- | --- | --- | --- | --- | --- | --- |
|  | Model 1 | | Model 2 | | Model 3 | |
|  | *Beta* | 95% C. I. | *Beta* | 95% C. I. | *Beta* | 95% C. I. |
| Intercept |  |  |  |  |  |  |
| Negative affectivity | 0.42 *** | 0.36 – 0.48 |  |  |  |  |
| Detachment | 0.17 *** | 0.11 – 0.22 |  |  |  |  |
| Antagonism | -0.03 | -0.10 – 0.03 |  |  |  |  |
| Disinhibition | 0.02 | -0.05 – 0.09 |  |  |  |  |
| Anankastia | 0.02 | -0.04 – 0.08 |  |  |  |  |
| Psychoticism | 0.19 *** | 0.12 – 0.26 |  |  |  |  |
| Behavioral inhibition |  |  | 0.38 *** | 0.31 – 0.45 |  |  |
| Fight-Flight-Freeze |  |  | 0.09 ** | 0.02 – 0.16 |  |  |
| Drive |  |  | 0.04 | -0.03 – 0.11 |  |  |
| Reward responsiveness |  |  | -0.16 *** | -0.24 – -0.09 |  |  |
| Fun seeking |  |  | 0.04 | -0.03 – 0.11 |  |  |
| IU - Limit. act. |  |  |  |  | 0.28 *** | 0.20 – 0.36 |
| IU - Burden |  |  |  |  | 0.39 *** | 0.30 – 0.47 |
| IU - Vigilance |  |  |  |  | -0.05 | -0.12 – 0.02 |
| *R^2^* | .42 |  | .19 |  | .36 |  |

Note. 95% C. I.: 95% confidence interval. IU – Limit. Act: Limited ability to act due to intolerance of uncertainty. IU – Burden: Burden due to intolerance of uncertainty. IU- Vigilance: Vigilance due to intolerance of uncertainty. R^2^: coefficient of determination; Delta R^2^: Difference in the coefficient of determination in the comparison with a model including only the control variables as predictors; *: *p* < .05; **: *p* < .01; ***: *p* < .001.

Table D. Regression analyses with the Health Anxiety scales as outcome

|  | Health anxiety | | | | | |
| --- | --- | --- | --- | --- | --- | --- |
|  | Model 1 | | Model 2 | | Model 3 | |
|  | *Beta* | 95% C. I. | *Beta* | 95% C. I. | *Beta* | 95% C. I. |
| Intercept |  |  |  |  |  |  |
| Negative affectivity | 0.42 *** | 0.36 – 0.48 |  |  |  |  |
| Detachment | 0.07 * | 0.01 – 0.13 |  |  |  |  |
| Antagonism | 0.06 | -0.01 – 0.12 |  |  |  |  |
| Disinhibition | 0.03 | -0.05 – 0.10 |  |  |  |  |
| Anankastia | 0.10 ** | 0.04 – 0.16 |  |  |  |  |
| Psychoticism | 0.09 * | 0.01 – 0.16 |  |  |  |  |
| Behavioral inhibition |  |  | 0.35 *** | 0.28 – 0.42 |  |  |
| Fight-Flight-Freeze |  |  | 0.12 ** | 0.05 – 0.19 |  |  |
| Drive |  |  | 0.06 | -0.02 – 0.13 |  |  |
| Reward responsiveness |  |  | -0.10 * | -0.18 – -0.02 |  |  |
| Fun seeking |  |  | 0.08 * | 0.02 – 0.15 |  |  |
| IU - Limit. act. |  |  |  |  | 0.19 *** | 0.10 – 0.27 |
| IU - Burden |  |  |  |  | 0.38 *** | 0.29 – 0.47 |
| IU - Vigilance |  |  |  |  | 0.03 | -0.04 – 0.10 |
| *R^2^* | .36 |  | .18 |  | .31 |  |

Note. 95% C. I.: 95% confidence interval. IU – Limit. Act: Limited ability to act due to intolerance of uncertainty. IU – Burden: Burden due to intolerance of uncertainty. IU- Vigilance: Vigilance due to intolerance of uncertainty. R^2^: coefficient of determination; Delta R^2^: Difference in the coefficient of determination in the comparison with a model including only the control variables as predictors; *: *p* < .05; **: *p* < .01; ***: *p* < .001.

Table E. Regression analyses with the Psychological factor scale of the C19-PS instrument as outcome

|  | **Psychological** | | | | | |
| --- | --- | --- | --- | --- | --- | --- |
|  | Model 1 | | Model 2 | | Model 3 | |
|  | *Beta* | 95 % C. I. | *Beta* | 95 % C. I. | *Beta* | 95 % C. I. |
| Intercept |  |  |  |  |  |  |
| Negative affectivity | 0.34 *** | 0.27 – 0.41 |  |  |  |  |
| Detachment | -0.03 | -0.09 – 0.04 |  |  |  |  |
| Antagonism | 0.05 | -0.03 – 0.12 |  |  |  |  |
| Disinhibition | 0.03 | -0.05 – 0.11 |  |  |  |  |
| Anankastia | 0.17 *** | 0.11 – 0.24 |  |  |  |  |
| Psychoticism | 0.01 | -0.07 – 0.09 |  |  |  |  |
| Behavioral inhibition |  |  | 0.30 *** | 0.22 – 0.37 |  |  |
| Fight-Flight-Freeze |  |  | 0.10 ** | 0.03 – 0.17 |  |  |
| Drive |  |  | 0.16 *** | 0.09 – 0.24 |  |  |
| Reward responsiveness |  |  | -0.05 | -0.13 – 0.03 |  |  |
| Fun seeking |  |  | 0.03 | -0.04 – 0.10 |  |  |
| IU - Limit. act. |  |  |  |  | 0.19 *** | 0.10 – 0.28 |
| IU - Burden |  |  |  |  | 0.27 *** | 0.17 – 0.36 |
| IU - Vigilance |  |  |  |  | 0.06 | -0.01 – 0.14 |
| *R^2^* | 0.22 |  | 0.15 |  | 0.23 |  |

Note. 95% C. I.: 95% confidence interval. IU – Limit. Act: Limited ability to act due to intolerance of uncertainty. IU – Burden: Burden due to intolerance of uncertainty. IU- Vigilance: Vigilance due to intolerance of uncertainty. R^2^: coefficient of determination; Delta R^2^: Difference in the coefficient of determination in the comparison with a model including only the control variables as predictors; *: *p* < .05; **: *p* < .01; ***: *p* < .001.

Table F. Regression analyses with the Psycho-somatic factor scale of the C19-PS instrument as outcome

|  | **Psycho-somatic** | | | | | |
| --- | --- | --- | --- | --- | --- | --- |
|  | Model 1 | | Model 2 | | Model 3 | |
|  | *Beta* | 95 % C. I. | *Beta* | 95 % C. I. | *Beta* | 95 % C. I. |
| Intercept |  |  |  |  |  |  |
| Negative affectivity | 0.20 *** | 0.13 – 0.27 |  |  |  |  |
| Detachment | 0.07 * | 0.00 – 0.13 |  |  |  |  |
| Antagonism | 0.13 *** | 0.06 – 0.21 |  |  |  |  |
| Disinhibition | 0.09 * | 0.01 – 0.17 |  |  |  |  |
| Anankastia | 0.06 | -0.01 – 0.12 |  |  |  |  |
| Psychoticism | 0.10 * | 0.02 – 0.18 |  |  |  |  |
| Behavioral inhibition |  |  | 0.28 *** | 0.20 – 0.35 |  |  |
| Fight-Flight-Freeze |  |  | -0.09 * | -0.17 – -0.01 |  |  |
| Drive |  |  | 0.13 *** | 0.05 – 0.21 |  |  |
| Reward responsiveness |  |  | -0.26 *** | -0.34 – -0.18 |  |  |
| Fun seeking |  |  | 0.10 ** | 0.03 – 0.18 |  |  |
| IU - Limit. act. |  |  |  |  | 0.32 *** | 0.22 – 0.41 |
| IU - Burden |  |  |  |  | 0.13 ** | 0.03 – 0.23 |
| IU - Vigilance |  |  |  |  | -0.05 | -0.13 – 0.03 |
| *R^2^* | 0.24 |  | 0.08 |  | 0.16 |  |

Note. 95% C. I.: 95% confidence interval. IU – Limit. Act: Limited ability to act due to intolerance of uncertainty. IU – Burden: Burden due to intolerance of uncertainty. IU- Vigilance: Vigilance due to intolerance of uncertainty. R^2^: coefficient of determination; Delta R^2^: Difference in the coefficient of determination in the comparison with a model including only the control variables as predictors; *: *p* < .05; **: *p* < .01; ***: *p* < .001.

Table G. Regression analyses with the Economic factor scale of the C19-PS instrument as outcome

|  | **Economic** | | | | | |
| --- | --- | --- | --- | --- | --- | --- |
|  | Model 1 | | Model 2 | | Model 3 | |
|  | *Beta* | 95 % C. I. | *Beta* | 95 % C. I. | *Beta* | 95 % C. I. |
| Intercept |  |  |  |  |  |  |
| Negative affectivity | 0.16 *** | 0.09 – 0.23 |  |  |  |  |
| Detachment | 0.04 | -0.02 – 0.11 |  |  |  |  |
| Antagonism | 0.15 *** | 0.07 – 0.22 |  |  |  |  |
| Disinhibition | <.001 | -0.08 – 0.08 |  |  |  |  |
| Anankastia | 0.14 *** | 0.07 – 0.21 |  |  |  |  |
| Psychoticism | 0.13 ** | 0.05 – 0.21 |  |  |  |  |
| Behavioral inhibition |  |  | 0.26 *** | 0.19 – 0.34 |  |  |
| Fight-Flight-Freeze |  |  | -0.11 ** | -0.18 – -0.03 |  |  |
| Drive |  |  | 0.13 ** | 0.05 – 0.21 |  |  |
| Reward responsiveness |  |  | -0.15 ** | -0.23 – -0.06 |  |  |
| Fun seeking |  |  | 0.05 | -0.03 – 0.12 |  |  |
| IU - Limit. act. |  |  |  |  | 0.22 *** | 0.12 – 0.31 |
| IU - Burden |  |  |  |  | 0.10 * | 0.00 – 0.21 |
| IU - Vigilance |  |  |  |  | 0.09 * | 0.01 – 0.17 |
| *R^2^* | 0.22 |  | 0.06 |  | 0.14 |  |

Note. 95% C. I.: 95% confidence interval. IU – Limit. Act: Limited ability to act due to intolerance of uncertainty. IU – Burden: Burden due to intolerance of uncertainty. IU- Vigilance: Vigilance due to intolerance of uncertainty. R^2^: coefficient of determination; Delta R^2^: Difference in the coefficient of determination in the comparison with a model including only the control variables as predictors; *: *p* < .05; **: *p* < .01; ***: *p* < .001.

Table H. Regression analyses with the Social factor scale of the C19-PS instrument as outcome

|  | **Social** | | | | | |
| --- | --- | --- | --- | --- | --- | --- |
|  | Model 1 | | Model 2 | | Model 3 | |
|  | *Beta* | 95 % C. I. | *Beta* | 95 % C. I. | *Beta* | 95 % C. I. |
| Intercept |  |  |  |  |  |  |
| Negative affectivity | 0.28 *** | 0.21 – 0.35 |  |  |  |  |
| Detachment | -0.01 | -0.08 – 0.06 |  |  |  |  |
| Antagonism | 0.11 ** | 0.03 – 0.18 |  |  |  |  |
| Disinhibition | -0.02 | -0.11 – 0.06 |  |  |  |  |
| Anankastia | 0.16 *** | 0.09 – 0.23 |  |  |  |  |
| Psychoticism | 0.05 | -0.04 – 0.13 |  |  |  |  |
| Behavioral inhibition |  |  | 0.28 *** | 0.21 – 0.36 |  |  |
| Fight-Flight-Freeze |  |  | 0.04 | -0.03 – 0.12 |  |  |
| Drive |  |  | 0.16 *** | 0.08 – 0.23 |  |  |
| Reward responsiveness |  |  | -0.04 | -0.12 – 0.04 |  |  |
| Fun seeking |  |  | 0.02 | -0.05 – 0.09 |  |  |
| IU - Limit. act. |  |  |  |  | 0.16 ** | 0.07 – 0.26 |
| IU - Burden |  |  |  |  | 0.22 *** | 0.12 – 0.32 |
| IU - Vigilance |  |  |  |  | 0.07 | -0.01 – 0.15 |
| *R^2^* | 0.20 |  | 0.12 |  | 0.17 |  |

Note. 95% C. I.: 95% confidence interval. IU – Limit. Act: Limited ability to act due to intolerance of uncertainty. IU – Burden: Burden due to intolerance of uncertainty. IU- Vigilance: Vigilance due to intolerance of uncertainty. R^2^: coefficient of determination; Delta R^2^: Difference in the coefficient of determination in the comparison with a model including only the control variables as predictors; *: *p* < .05; **: *p* < .01; ***: *p* < .001.
